# Differentiated Data Aggregation Routing Scheme for Energy Conserving and Delay Sensitive Wireless Sensor Networks

**DOI:** 10.3390/s18072349

**Published:** 2018-07-19

**Authors:** Xujing Li, Wei Liu, Mande Xie, Anfeng Liu, Ming Zhao, Neal N. Xiong, Miao Zhao, Wan Dai

**Affiliations:** 1 School of Information Science and Engineering, Central South University, Changsha 410083, China; xujingli@csu.edu.cn (X.L.); afengliu@mail.csu.edu.cn (A.L.); 2 School of Informatics, Hunan University of Chinese Medicine, Changsha 410208, China; weiliu@csu.edu.cn; 3 School of Computer Science and Information Engineering, Zhejiang Gongshang University, Hangzhou 310018, China; 4 The State Key Laboratory of Industrial Control Technology, Zhejiang University, Hangzhou 310027, China; 5 School of Software, Central South University, Changsha 410075, China; meanzhao@mail.csu.edu.cn; 6 Department of Mathematics and Computer Science, Northeastern State University, Tahlequah, OK 74464, USA; xiongnaixue@gmail.com; 7 State Grid Hunan Electric Power Company Limited Research Institute, Changsha 410007, China; terrence3333@163.com (M.Z.); pengt18@163.com (W.D.)

**Keywords:** differentiated data aggregation routing, wireless sensor networks, delay sensitive, energy efficiency

## Abstract

Data aggregation is a widely adopted method to effectively reduce the data transmission volume and improve the lifetime of wireless sensor networks (WSNs). In the data aggregation networks, some parameters directly determine the delay of aggregation. In industrial applications, the data generated by different sensors have different requirements for delay or other QoS performance. In the previous study, a common strategy is that all kinds of data is aggregated into one frame when the condition is satisfied with a QoS requirement, which causes excessive energy consumption and severely impairs the lifetime of network. A Differentiated Data Aggregation Routing (DDAR) scheme is proposed to reduce energy consumption and guarantee that the delay could be controlled within the corresponding QoS requirement constraint. The primary contributions of the DDAR scheme are the following: (a) The DDAR scheme makes data with different QoS requirement route to the sink along the different paths. The parameters of the aggregators in each path, such as aggregation deadline ($T_{t}$) and the aggregation threshold ($N_{t}$), are configured according to the QoS requirements. Accordingly, energy consumption can be reduced without degrading the performance of data transmission. (b) Based on DDAR scheme, an improved DDAR scheme is proposed to further improve performance through fully utilize the residual energy in the nodes which are far from the sink. The frequency of aggregation of these nodes increases by reducing the value of $T_{t}$ and $N_{t}$ so as to further improve the energy efficiency and reduce delay. Simulation results demonstrate that compared with the previous scheme, this scheme reduces the delay by 25.01%, improves the lifetime by 55.45%, and increases energy efficiency by 83.99%. The improved DDAR scheme improves the energy efficiency by 33.97% and service guarantee rate by 10.11%.

## 1. Introduction

Industrial intelligent technology has attracted the considerable attention of the manufacturing industries of all countries in the world. Industry and academia have invested a large number of funds, technology, and efforts in this area. Its main objective is to digitize and intelligentize the information of supplies, manufacturing and sale information by cyber-physical systems (CPS), and achieve a fast, efficient and personalized product supply [1,2]. Smart industrial wireless sensor networks (SIWSNs) is identified as an essential technology to pave the way to this goal [2,3,4]. With SIWSNs, data sensing, gathering and communicating are performed intelligently by all kinds of industrial wireless sensors (e.g., photoelectric sensor, ultrasonic sensor, gas sensor, video sensor [5,6,7,8]). Thus, data can be exchanged and managed autonomously and efficiently [9,10]. Additionally, sensors can self-organize the routing path in the network [11,12,13] and automatically update the path according to the application. This feature gives SIWSNs a superior performance that the inherent drawbacks of wired industrial networks can be well overcome [14,15]. Nowadays, smart WSNs have been widely applied in industrial application scenarios, ranging from environment monitoring to urban health monitoring [14,16], vehicular communication networks [6,17], cyber-physical cloud systems [18,19,20,21,22], multi-channel cognitive radio networks [8,23], crowdsourcing networks [24,25,26], social network [27,28,29,30]. The growing demand for wireless sensors in the smart industry makes Quality-of-Service (QoS) one of the paramount issues in wireless sensor-based applications [31,32,33,34].

For SIWSNs, the two most significant QoS indicators are the energy efficiency [11,15,21,23,33,35,36,37,38] and delay [2,8,10,33,35,37,39,40]. Delay is vital for industrial sensor networks. The studies in energy efficiency focus on how to reduce the energy consumption to improve network lifetime. For the optimization of energy consumption, data aggregation is an effective method [28,40,41]. In the data transmission process, data packets can be aggregated into the packets with a smaller size. Therefore, energy consumption can be reduced [40,41,42]. This is categorized as aggregation routing problem.

In the previous research of data aggregation routing, all nodes have the same values of $N_{t}$ and $T_{t}$. Small value of $N_{t}$ and $T_{t}$ are set to make the data with small delay deadline satisfy its QoS requirement. However, this keeps a low network lifetime. In most industrial applications, most of the data is delay-insensitive, and there is small proportion of delay-sensitive data. Adopting the top QoS parameters setting (setting $N_{t}$ and $T_{t}$ to satisfy the QoS requirement of the most emergent data) can ensure that all the data meet their requirements of application, but the frequency of aggregation is improved and the lifetime is significantly reduced.

In this scenario, differentiated data aggregation routing is a wise option [43]. The main idea is to set different dominant parameters for different QoS requirements. Based on the premise of meeting different QoS requirements, the energy consumption and delay are reduced and the performances increase. However, the special nature of data aggregation routing makes the scheme difficult to be fully implemented. Each node in the network prepares various $N_{t}$ and $T_{t}$ for QoS requirements. When the data in a node satisfies some aggregation condition, all data packets in the queue should be aggregated and transferred. However, this strategy makes the data with restrict QoS requirement difficult to be keep valid, and gives the data with loose QoS requirement an unnecessary and superfluous service guarantee. Moreover, if we use the strategy that only the data with corresponding service requirement is aggregated when a requirement is satisfied, the data storage structure is more complex and the energy consumption in transmission increases. In summary, the strategy only setting different $N_{t}$ and $T_{t}$ for corresponding QoS requirements has a very limited optimization performance on Diffserv networks, proposing an efficient differentiated services strategy in WSNs is a challenging issue.

For the above problems, a Differentiated Data Aggregation Routing (DDAR) scheme is proposed to reduce energy consumption and ensure that all kinds of data meet their service requirements. Salient features of the proposed work are given as follows:

- The DDAR scheme is a novel data aggregation routing framework. In this framework, each node configures only one set of parameters to satisfy a certain QoS requirement. When a node performs aggregation, it searches an aggregator whose service most closely matches its QoS requirement for next hop. The most closely matching refers to the nodes which have the smallest difference of QoS requirement with the sender. DDAR scheme ameliorates the high energy consumption, complex storage and poor service guarantee in previous strategies. Thus, DDAR scheme realizes the differentiated data aggregation routing in the true sense, and is able to significantly reduce energy consumption while ensuring that data transmission of data packets meets service requirement.
- Based on DDAR scheme, we propose an improved DDAR scheme to reduce delay and improve energy efficiency by utilizing the residual energy in the nodes far from the sink. Whatever routing strategy is adopted, the data volume a node transmits decreases with the increase of distance to the sink. This phenomenon illustrates that the energy consumption of the nodes near the sink is larger than the nodes far from the sink, there is residual energy in the nodes when the network dies. In this paper, improved DDAR enhances the performance by increasing the frequency of aggregation.
- In this paper, we propose the differentiated data aggregation routing scheme. Simulation results demonstrate that DDAR can improve the service guarantee rate by 25.1%, network lifetime by 55.45% and energy efficiency by 83.99%.

The rest of this paper is organized as follows: in Section 2, related works are reviewed. The system model and problem statement are described in Section 3. In Section 4, design details on DDAR scheme are presented. Section 5 is simulation results and comparisons for the optimization performance of DDAR scheme. We conclude in Section 6. 

## 2. Related Work

With the rapid development of microprocessor technology, sensors have achieved considerable progress [44,45,46]. At present, sensors have been widely used in various aspects of human life [47,48,49], especially in the industrial field. In industrial production applications, the most two noteworthy indicators are energy efficiency and the timeliness in data transmission (i.e., delay) [50,51]. Therefore, reducing the energy consumption in the network, prolonging the network lifetime while ensuring the data transmission delay satisfies the service requirement is a significant and challenging issue in the research field [52,53].

### 2.1. Research on Data Aggregation Routing

Improving the energy efficiency is a valuable research field [11,15,21,23,33,35,36,37,38]. Data aggregation is a method to effectively reduce the data volume in the data transmission process [54,55] and improve the lifetime of the network. Also, other performances of the network, such as the probability of the collision, can be optimized [56]. Thus, plenty attention and effort have been put into this field [57]. There are different data aggregation methods for different data aggregation ratios. The methods based on the max(), min(), avg() functions have the highest data aggregation degree. Such methods aggregate n data packets into 1 data packet, which can be abstracted as a convergecast problem [41,58]. Convergecast are divided into two phases. In the first phase, what nodes do is to produce data packets. In the second phase, nodes determine whether to send data. If a node sends data, all data packets in the node are aggregated into one data packet. In [41], Xu et al. propose an algorithm and prove the delay of their scheme is at most 16R+Δ−14 time slots. In [58], Huang et al.’s algorithm has an upper bound of 23R+Δ−18 time slots in delay, where R is the network radius and Δ is the maximum node degree.

Convergecast refers to a particular case of data aggregation in which each node sends only one data packet during the data transmission process. The networks with an aggregation ratio between 0 to 1 are normal [59], the aggregation conforms to Equation (1):

(1) $$ℒ\left(d_{i},d_{j}\right)=Max\left(l\left(d_{i}\right)+l\left(d_{j}\right)\right)+(1-C_{i,j})*Min\left(l\left(d_{i}\right),l\left(d_{j}\right)\right)$$

$d_{i},d_{j}$ is data packets queue i and j. $l\left(d_{i}\right)$ refers to the length before aggregation. $C_{i,j}$ is the correlation coefficient between nodes $d_{i}$ and $d_{j}$, which is between 0 to 1. The value of $C_{i,j}$ is inversely proportional to distance of the two nodes [59]. $ℒ\left(d_{i},d_{j}\right)$ is the length of data packets queue after the aggregation of $d_{i}$ and $d_{j}$. Unlike Convergecast, the generation of data in this kind of networks is not periodical. The time data packets received by a node is uncertain, this increases the difficulty of designing a data aggregation routing scheme. The nodes in this scenario keep a principle for data aggregation in advance, that is an upper bound of the length of the data queue and the time packets waits in a node. If the length exceeds or the time expires, the node performs aggregation. These two bounds are described as aggregation threshold $N_{t}$ and aggregation timer $T_{t}$. The average aggregation delay linearly increases as the aggregation timer or the aggregation threshold increases [42]. In AAR scheme proposed in [40], the waiting time in nodes is significantly reduced and the aggregating and transmitting load are balanced.

The optimization strategies above are intended to optimize the routing path, not to optimize data aggregation. Data aggregation is efficient in the case that as many packets as possible perform aggregation. Villa et al. [60] proposed a DRINA strategy which improves the probability of packets routing along the same path. Traditional routing methods (e.g., shortest routing method) makes data packets route along many routing paths scattered in the network, which reduces the number of data packets routing in the same path. DRINA constrains the routing path generation so that the data packets can only reach the sink along specific few paths. The number of data which routes along the same path can be significantly improved, the network delay can be reduced, and the lifetime can also be improved. A modified minimum hop routing strategy is adopted in DRINA. Each node selects the node with the minimum “hop” as next hop. In the initialization, “hop” is the number of hops to the sink. Once data packets reach the sink via a path, the nodes on the path set their “hop” to 0, and update their route, then their neighbor nodes update their route. Thereby, the part which connects to the sink of many routing paths coincides with the path on which the “hop” of the nodes are set to 0, the number of the connections to the sink significantly decreases.

Cluster-based WSNs is data aggregation network. In a cluster network, the nodes are divided into several clusters. A cluster consists of two types of nodes, cluster heads (CHs) and cluster member nodes. CHs collect the data packets generated in their cluster and aggregate the data and transmit it to next hop (possibly another CH). The relevant studies can be found in Ref. [61].

The research above are all classical data aggregation routing methods. There is another method based on approximate data aggregation. In our previous work, we proposed a routing method combining approximate data aggregation method with traditional data aggregation method [61]. In some applications, such as monitoring the temperature and humidity of plants on the farm, the monitored value is allowed within a certain error range. Thus, the monitoring value can be set as the value of a representative node, so it is unnecessary to transmit all the monitoring value of each node to the sink [62]. The data transmission volume of the n nodes’ network can be reduced to 1/n. The key to this method is to elect the representative node. A simple idea is to combine a certain node (representative node) to its neighbor nodes whose monitoring values is in the error range into a set. After a round of combination, the set can be expanded to the neighbor nodes of the newly included nodes in the set. When the set stops expanding, the set of representative node establishes. All nodes in this set can be represented by the representative node. In [62], we propose a scheme to improve data aggregation along the path representative nodes route to the sink. Table 1 is presented to summarize the references:

### 2.2. Research on Delay Optimization

Generally, the delay in WSNs refers to the difference between the time when the data is generated and the time data reaches the sink. There is various research which aims to reduce delay. Based on the layer the optimization methods work in, the research can be categorized to the optimization methods in the MAC layer, network layer, transport layer and application layer. Moreover, cross-layer optimization methods have been studied and proposed. In the methods which work in the MAC layer, a critical performance indicator which is related to delay is data reception ratio [16]. In WSNs, the distance the data can be transmitted and the reception ratio is positively related to the node’s transmission power. It is a feasible method to optimize delay by optimizing the transmission power. In [16], the relationship between transmission power and data reception ratio is shown.

There is quite a bit of research on optimizing delay in the network layer. For example, in the shortest routing scheme, each node chooses the node closest node to the target as the next hop each time. It is a delay-efficient routing algorithm. However, in some WSNs, it may cause excessive energy consumption to some key nodes on the shortest routing path. The delay and energy consumption should be taken into account in routing algorithms to balance the energy and improve the overall network performance. This comprehensive performance optimization strategy can be seen in [20,36,38,39,57]. Besides, network attacks exist in wireless networks, delay routing optimizations including security factors become more complicated. To prevent the attacks, the data packets are transmitted on multiple routing paths to the sink. In this policy, the sink may receive multiple identical data packets [36]. Although the energy consumption in the strategy is more than that of single data packet routing strategy, the delay can be reduced as the delay is the time difference which is determined by the first data packet reach the sink. In Ref. [63], SDER scheme is proposed. Each data packet splits into m slices, all slices are routed along the different paths. A data packet can be restored by n slices. It’s difficult for attackers to catch n slices, so the security can be improved. This is a compromise between energy consumption and security. Compared to multiple data packets transmitted to the sink, this scheme consumes less energy and performs better regarding security. However, there is no advantage for the delay as n slices received by the sink is considered as success transmission. In duty cycle based WSNs, nodes periodically switch between the sleep state and the awake state. When the sender has packets to send, the node closest to the sink in the forwarding node set may sleep and cannot receive packets. The senders has two options, waiting for that closest node or selecting the closet awake node. Which option causes more delay can’t be judged. A dynamic routing algorithm is proposed in [64] to optimize the delay in duty cycle WSNs. Additionally, an optimization method is proposed in [39]. The main idea is that, the sender sends data to multiple receivers. Data received by a receiver is regarded as the successful transmission. This method is able to decrease the probability of retransmission, so delay decreases. 

Because the reliability of data transmission in the wireless transmission environment is much lower than that of the wired network, the strategy to guarantee the transmission reliability of data also has excellent optimization effect on delay. The most commonly used is the send-wait retransmission mechanism. In this mechanism, after sender sends a data packet, it waits for the receiver to reply an ACK confirming the reception of the data packet. The receiver is considered not to receive the packet when the sender does not receive the ACK after a stipulated period. If so, the sender resends the packet. The process of sending a packet continues until the sender receives the ACK, or the number of retransmissions reach the predetermined threshold. This method brings much delay in the networks with high data bit error rate. The study in [65] improves these methods.

Differentiated service is another effective method to guarantee the QoS in applications [43]. In differentiated services, nodes use different routing methods for data packets with different QoS requirements. For strict service requirement (e.g., small delay) packets, they should be forwarded immediately, and for loose requirement packets, delayed forwarding is adopted to save energy. Although this method can be adopted in many applications, as we mentioned earlier, the implementation of efficient differentiated services in data aggregation is still a great challenge. We summarizes these issues in Table 2. 

## 3. System Model and Problem Statement

### 3.1. System Model

The system model in this paper can be abstracted as a planar wireless network. All nodes are randomly deployed in a two-dimensional circular space centered on the sink. There are two types of nodes in the network, aggregators and sensors, which are heterogeneous in energy and function. The aggregators collect data packets generated by nearby sensors, aggregate and transmit data packets as well. Sensors send the generated data packets to a nearby aggregator.

Since the aggregators have a limit of the transmission distance, most of the data packets need multiple hops to reach the sink. The number of hops for data packets decreases with the decrease of the distance to the sink. Depending on the data transmission path, the model can be regarded as a network with a fixed tree topology; each node in the network is in a fixed logical level. The next hop of an aggregator is called the aggregator in the upper layer of that aggregator.

The data packets in the network have distinct service, and the delay that the data packets can tolerate is determined by its service. If the delay exceeds the corresponding bound, the data becomes invalidated.

**Figure 1:**
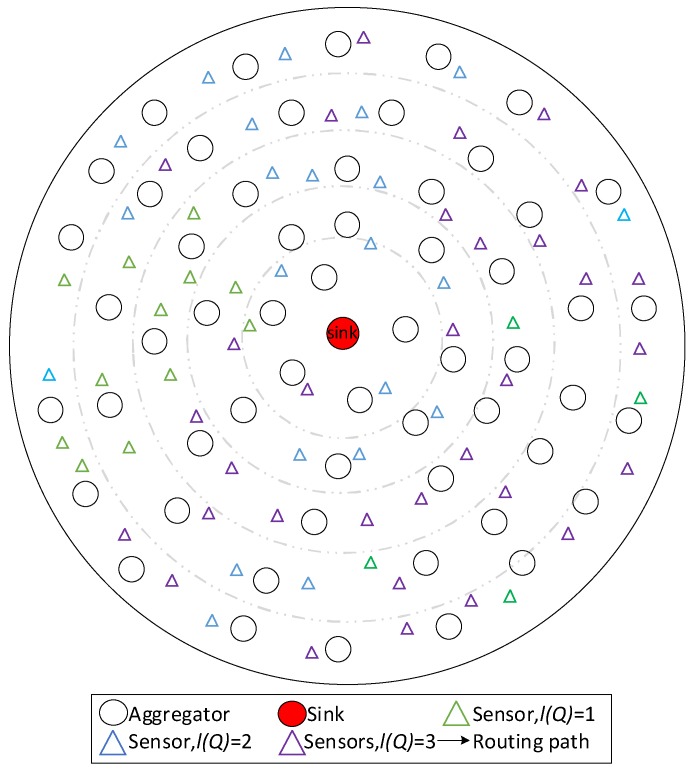
An instance of the system model.

The system network is Diffserv network; an example is provided below. As seen in Figure 1, the network contains one sink, 57 aggregators and 70 sensors. There are three services in the network.

All the aggregators cycle in two phases. One is data generation phase in which aggregators receive the data packets generated by nearby sensors. The other is data transmission phase. In this phase, the aggregators determine whether the packets in the waiting queue meet the standard of aggregation. When the aggregation condition is satisfied, aggregation and transmission are carried out. There is no data received in this phase. Sensors have two phases in a cycle as well. In data generation phase, sensors produce data and transmit data to an aggregator. At most one data packet can be produced by a sensor during a cycle. Sensors sleep when aggregators are in data transmission phase. The two cycles are same in time.

The system model can be abstracted as a network of tree topology, the root is the sink. The number of the hops to the sink determines the layer that the nodes are in. In this network, the operation of sensors is consistent and synchronized, the time of each period of aggregators is fixed, and the operation of aggregators in the same layer is synchronized. The operation of aggregators in the different layers is slightly different in time so that an aggregator could judge aggregation immediately after receiving the data queues from other aggregators. The unit time is the time that a cycle costs.

### 3.2. System Parameters

**Definition** **1.** *Probability of Generates a Packet.*

In the network, the average frequency at which data packet is generated in a cycle by a node can be considered as the probability that a sensor generates a packet in a cycle; this can be defined as the probability of generating a packet $P_{\alpha }$. In the model, all the sensors have the same value of $P_{\alpha }$.

**Definition** **2.** *Data Aggregation Ratio.*

**Figure 2:**
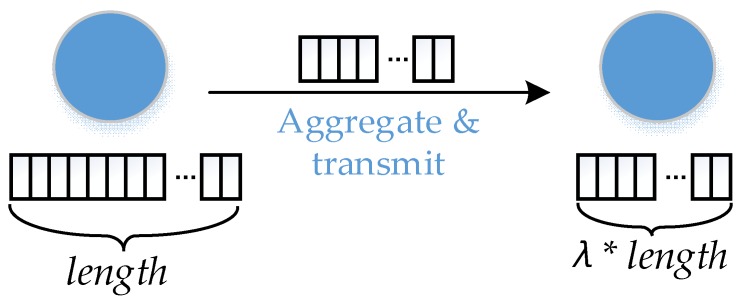
Aggregation reduces the length of the aggregation queue.

In the process of data transmission phase, the redundant data is eliminated. The length of the actual data packet queue in transmission process is smaller than the length of the queue before aggregation (see from Figure 2). Data aggregation ratio λ is defined as the ratio of that two lengths. In this model, all the aggregators have the same value of λ.

**Definition** **3.** *Packet Aggregation Threshold.*

**Figure 3:**
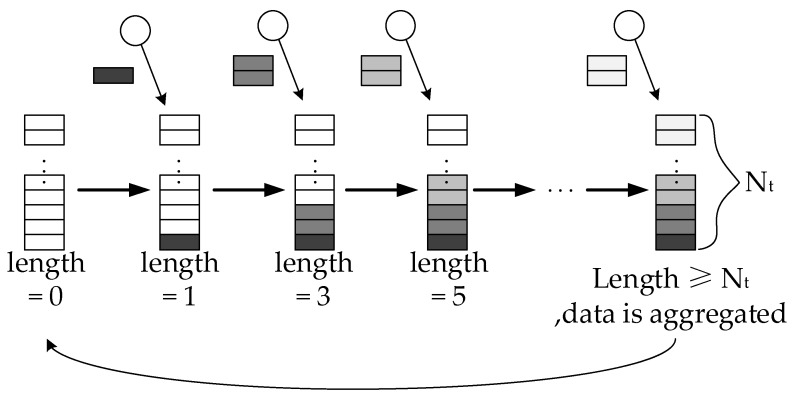
Packet aggregation occurs since the length of packet queue is equal to or greater than $N_{t}$.

The aggregators use a data waiting queue to store the arriving data packets. If the length of the queued packets is greater than or equal to the predetermined aggregation threshold, the aggregators aggregate and send data (see from Figure 3). The packet aggregation threshold $N_{t}$ is defined as the maximum of the length of the waiting queue in an aggregator.

**Definition** **4.** *Value of the Packet Aggregation Timer.*

**Figure 4:**
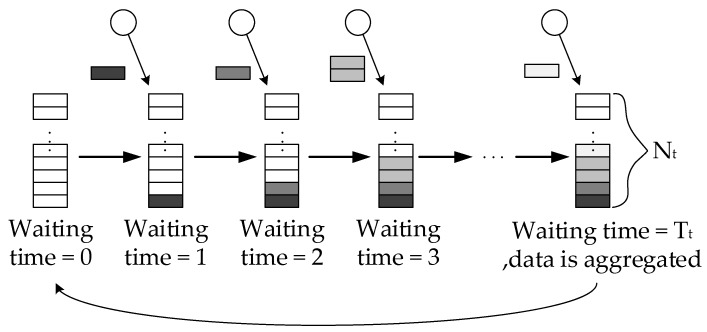
Packet aggregation occurs since the value of the aggregation timer is equal to $T_{t}$.

The data in WSNs has real-time nature, the delay of data from generation to arrive to the sink should be limited within the acceptable latency range. In each aggregator, there is a timer, which represents the time difference from the last aggregation. The large value of timer means that the data packets in the queue have long average waiting time, and the data is more likely to be invalidated. The aggregation timer $T_{t}$ refers to the maximum allowable time to wait for an aggregation (see from Figure 4). As long as the value of the timer is equivalent to the predetermined $T_{t}$, the queue would be aggregated and transmitted.

**Definition** **5.** *Service Requirement.*

In different scenarios, data tolerates a different degree of latency. The data in fire monitoring system needs a high real-time guarantee, while there is no need for temperature monitoring system to have the high real-time ability. Service requirement Q is defined as the longest acceptable delay of a data packet during the transmission process. Once the delay of data exceeds the corresponding service requirement $Q^{i}$, the data is considered to be invalidated. In Diffserv networks, the number of service requirements is more than one. For each sensor in the network, the service requirement of data packets generated by a sensor is constant. In this system, service requirement is randomly assigned to each node.

**Definition** **6.** *Service Tag.*

The service tag is a tag that the aggregators determine at initialization to match the sensors with corresponding service requirement. In the initialization process, the aggregators determine their own tags S based on service requirement of the nearby sensors. Based on the service tag of each aggregator, sensors determine which aggregator the data packets are transmitted to.

The parameters used in this paper are listed in Table 3.

### 3.3. Problem Statements

(1) Maximize service guarantee rate

To guarantee the real-time and validity of data, data should be transmitted to the sink as soon as possible. However, the energy in aggregators is limited; it is a practical and feasible method to restrict the delay of every packet according to the corresponding service requirement to ensure to be valid. Assume the number of valid data packets of each service requirement is $d_{i}$, the sum of data packets is D, maximize the service guarantee rate ρ is to maximize the expectation of $\sum_{i=1}^{Q}{𝒹}_{i}$/D:

(2) Max(ρ)=Max(∑i=1Q𝒹iD).

(2) Maximize energy efficiency

Energy efficiency is the ratio of the total energy consumed by the network to the sum of initial energy of each aggregator. In the model, all aggregators have the same energy $E_{INI}$. Assume the energy consumed by a node σ when the network stops working is $\gamma_{\sigma }$ and $E_{INI}$ is considered the initial energy of each node, the formula to maximize the energy efficiency Ξ can be expressed by the following equation:

(3) $$Max\left(\Xi \right)=\mathrm{Max}\frac{{\sum^{\text{​}}}_{i=1}^{N}\gamma_{i}}{{\sum^{\text{​}}}_{i=1}^{N}E_{INI}}$$

(3) Maximize network lifetime

Network lifetime refers to the time that the first node’s death occurs in the network [40,43]. When the energy in a node is used up, the network dies. Therefore, the length of network lifetime depends on the node which first runs out of the energy. Assuming that the energy consumption of aggregator σ in a unit time is $\xi_{\sigma }$, the initial energy is $E_{INI}^{i}$. To maximize the lifetime of the network is to maximize the network lifetime of the aggregator with the fastest energy consumption in the network. Therefore, Equation (4) can be obtained:

(4) $$\mathrm{Max}\left(ℒ\right)=Max\left(\underset{1\leq i\leq N}{\mathrm{Min}}\left(\xi_{i}/E_{INI}^{i}\right)\right).$$

In summary, the research objectives of the scheme are as follows:

(5) {Max(ρ)=Max(∑i=1Q𝒹iD)Max(Ξ)=Max∑​i=1Nγi∑​i=1NEINIMax(ℒ)=Max(Min1≤i≤N(ξi/EINIi)).

## 4. Optimization Mechanism Design

### 4.1. Research Motivation

**Figure 5:**
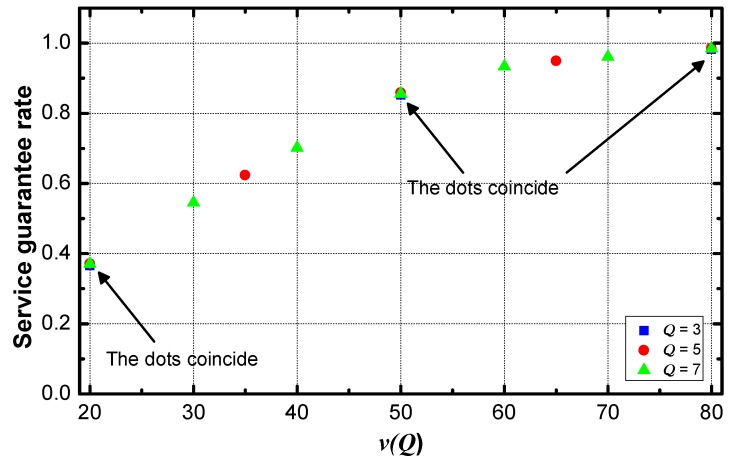
ρ vs. different value of service setting.

1. In the DiffServ networks without a method for service guarantee, there is a gap between service guarantee rates of different service requirements (shown in Figure 5). With the increase of $v\left(Q_{\varepsilon }\right)$, the service requirement becomes loose. The service guarantee rate is high when the service requirement is loose. That is, the rate increases with the increase of the value of service requirement. Therefore, it is necessary to reduce the transmission delay of data with a small value of service requirement to improve the service guarantee rate of these kinds of data.
2. Compared with the data generated near the sink, the data packets generated by sensing nodes far from the sink spend more hops to arrive at the sink. This process contributes most of the delay. The delay can be effectively reduced by reducing the delay of these packets, and service guarantee rate can be improved as well.
3. The aggregators far from the sink transmit fewer data packets than aggregators near the sink. The data aggregation and transmission are the primary consumption methods of energy. In the model, all the aggregators are homogenous in energy. To enhance the transmission frequency of these distant nodes, the energy efficiency can be improved while the lifetime doesn’t extensively deteriorate.

### 4.2. General Design of DDAR

The service requirement for data packets generated by each sensor is fixed, the service requirement of data can be directly mapped to the sensor. In DDAR, aggregators determine their service tag according to the number of service requirements of the nearby sensors. The value of $N_{t}$ and $T_{t}$ are calculated based on that tag. Then sensors select an aggregator as its data packets’ destination. Meanwhile, each aggregator establishes a next-hop route in accordance with the tag. DDAR can be expressed as follows:

- Aggregators identify their service tag.
- Aggregators configure $N_{t}$ and $T_{t}$.
- Sensors determine the destination of their data.
- Aggregators choose an aggregator with the same service tag as their next hop.
- Service tags rotate.

*Phase 1:* Configuration of service tag

Each aggregator identifies its service tag in this phase. The service requirements of all sensors in the pre-set range are counted, and the most frequent requirement is set as its service tag. If no sensor is near the aggregator, the aggregator determines its tag in phase 4.

**Figure 6:**
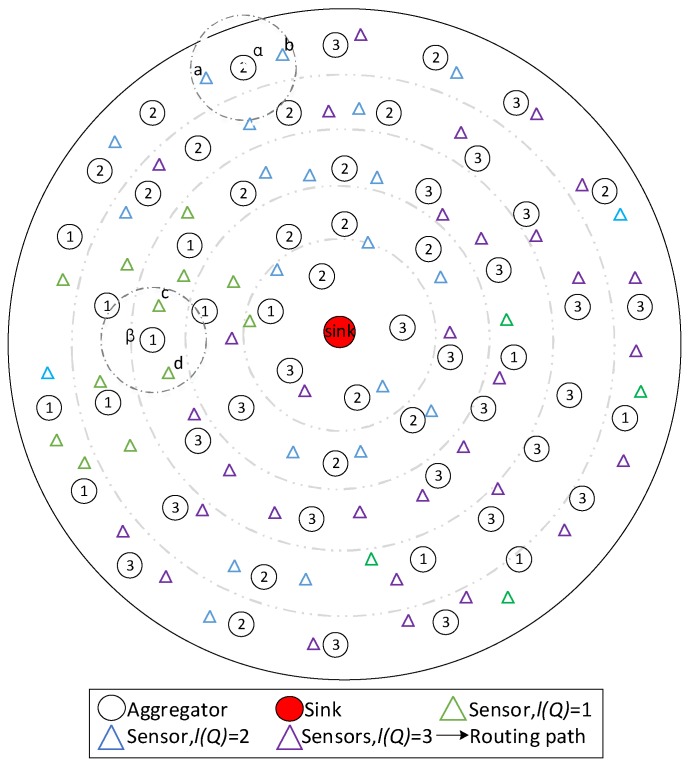
The sample operations in phase 1 of DDAR.

In Figure 6, aggregator α communicates with the sensors a and b. As the requirement of these sensors is level 2, the tag of α is set as 2. The sensors in aggregator β’s data collection range need level 1 service, β’s tag is set as 1.

The process can be described by Algorithm 1.
**Algorithm 1** Configuration of Service Tag of Aggregator σ.1: Aggregator σ sends a broadcast message to sensors in its predetermined communication range to inquire the rank of service of each node. 2: Each sensor replies a message to inform the rank of service $Q^{i}$ to the aggregator. 3: **For** each $Q_{\varepsilon }$ received by the aggregator **do** 4:    $q\left[l\left(Q_{\varepsilon }\right)\right]$++ 5: **End for** 6: maxIndex = 0 7: **For** each $q\left[l\left(Q_{\varepsilon }\right)\right]$
**do** 8: **If**
$q\left[l\left(Q_{\varepsilon }\right)\right]>q\left[\mathrm{maxIndex}\right]$
**then** 9:    maxIndex = $\left[l\left(Q_{\varepsilon }\right)\right]$ 10: **End if** 11: $S_{\sigma }$ = maxIndex


*Phase 2:* Configuration of $N_{t}$ and $T_{t}$

The parameters which dominate aggregation are aggregation timer and aggregation threshold, the average aggregation delay linearly increases as the aggregation timer or the aggregation threshold increases but is saturated at sufficiently large values of the aggregation timer or the aggregation threshold. After the identification of service tag, $N_{t}$ and $T_{t}$ are calculated. We design a formula for aggregators to configure $N_{t}$ and $T_{t}$ according to the tag, ensuring that the data service guarantee rates are suitable for comparison. The aggregators without tag don’t configure $N_{t}$ and $T_{t}$ until they have a tag.

The configuration formula of $N_{t}$ and $T_{t}$ is as follows (the formula of $T_{t}$ is same as that of $N_{t}$).

(6) $$N_{t}^{i}=0.5*v(Q^{i})*\frac{1}{\eta }*P_{\alpha }*\lambda^{0.5}$$

*Phase 3:* Finding the corresponding aggregator

Because of sensors’ limited power, sensed data needs to be transmitted to the sink via aggregators. In this phase, sensors choose the aggregators which their data reaches. After the aggregators determine their service tag, each sensor broadcasts to all surrounding aggregators in sensors’ data transmission range to inquire about their service tags. The requirement matching aggregator with the shortest distance is considered as the destination of the sensor’s data transmission. If none, the sensor would from bad to good selects an aggregator with better service as its data’s destination. If there is no node with a better service, the sensor from good to bad selects an aggregator with worse service.

**Figure 7:**
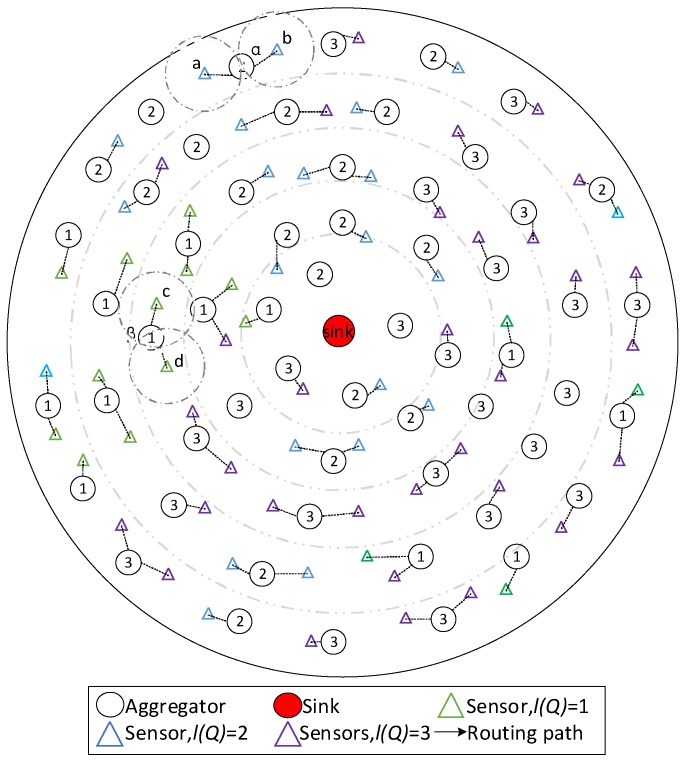
The sample operations in phase 3.

In Figure 7, aggregators in the data transmission range of a, b and c, d are α and β separately. The tag of α is same as the requirement of a and b, and the tag of β is same as the requirement of c and d. Therefore, a and b select α as their data’s destination, c and d select β as the destination.

The process can be described by Algorithm 2.
**Algorithm 2** Establishment of Routing for a Sensor ε.1: Sensor ε sends a broadcast message to aggregators in its predetermined communication range to inquire the tag of service of each node. 2: Each aggregator replies a message to inform the tag of service $S_{\sigma }$ to the sensor. 3: **For** each $S_{\sigma }$ received by the senisng node **do** 4:    **If**
$l\left(Q_{\varepsilon }\right)$ == $S_{\sigma }$
**then** 5:        Dest(ε)=σ 6:        **Return** 7:    **End if** 8: **End for** 9: **If** there is no aggregator whose $S_{\sigma }$ is equal to $l\left(Q_{\varepsilon }\right)$ 10:    $\mathrm{index}=l\left(Q_{\varepsilon }\right)-1$ 11:    **While**
index>0
**do** 12:        **For** each $S_{\sigma }$
**do** 13:            **If**
$S_{\sigma }==\mathrm{index}$
**then** 14:                Dest(ε)=σ 15:                **Return** 16:            **End if** 17:        **End for** 18:        index-- 19:    **End while** 20:    $\mathrm{index}=l\left(Q_{\varepsilon }\right)+1$ 21:    **While**
index≤Q
**do** 22:        **For** each $S_{\sigma }$
**do** 23:            **If**
$S_{\sigma }==\mathrm{index}$
**then** 24:                Dest(ε)=σ 25:                **Return** 26:            **End if** 27:        **End for** 28:        index++ 29:    **End while** 30: **End if**


The explicative remarks about Algorithm 2 are shown below:

Lines 3–8: Sensor searches an aggregator whose service tag is equal to the level of the sensor’s service requirements.

Lines 11–19: If there is no aggregator owning the corresponding service tag, the sensor searches an aggregator which has a better service from bad to good.

Lines 20–29: If there is no aggregator with no corresponding service or better, the sensor starts to find an aggregator with a worse service from good to bad.

The complexity analysis is described below:

The number of the aggregators in the network is set as N. the time that sensor broadcasts and receives message is O(1). Then the sensor selects an aggregator with the corresponding tag. The overhead of this step is O(N). If there is no such an aggregator, the sensor would go through the tags twice at most. Therefore, the time complexity of a sensor selects an aggregator is as Equation (7):

(7) O(1)+O(N)+O(N)+O(N)=O(N)

*Phase 4:* Finding the next hop of data packet queue

While sensors choose their data transmission aggregators, aggregators issue service tag queries to the surrounding aggregators in the upper layer (closer to the sink) to determine the next hop route. The candidate aggregators with no tag are informed to set the tag when there is no aggregator has the same tag. Then that node set $N_{t}$ and $T_{t}$ using Equation (6).

If there is still no aggregator with the same tag, the aggregator would from bad to good select an aggregator with a better service. If there is no aggregator with a better service, the aggregator selects a node with a worse service from good to bad. If the sink is in the predetermined range, the aggregator selects the sink as next hop.

**Figure 8:**
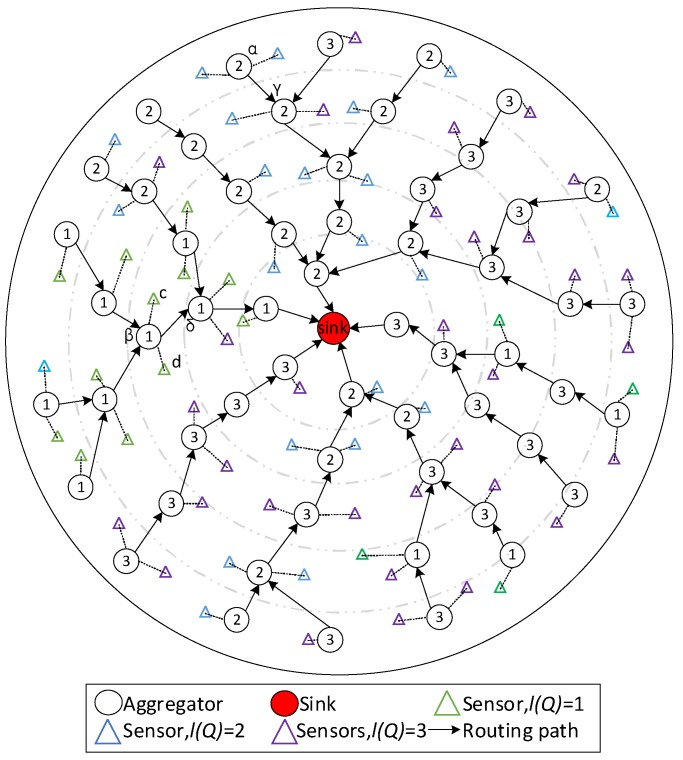
The sample operations in phase 4.

As shown in Figure 8, the tag of α is 1, in its neighbor aggregators, γ‘s tag is same as α. α chooses γ as its next hop. Same reasoning, β regards δ as its next hop.

The details can be described as the following algorithm.
**Algorithm 3** Establishment of Routing for an Aggregator in Level i
$\sigma^{i}$.1: Aggregator $\sigma^{i}$ sends a broadcast message to aggregators in its predetermined communication range to inquire the tag of service of each node. 2: Each aggregator replies a message to inform the tag of service $S_{\sigma^{i-1}}$ to the sensor. 3: **For** each $S_{\sigma^{i-1}}$ received by the senisng node **do** 4:    **If**
$S_{\sigma^{i}}$ == $S_{\sigma^{i-1}}$
**then** 5:        $Dest\left(\sigma^{i}\right)$ = $\sigma^{i-1}$ 6:        **Return** 7:    **End if** 8: **End for** 9: **If** there is an aggregator with no service tag 10:    $S_{\sigma^{i}}$ = $S_{\sigma^{i-1}}$ 11:    $Dest\left(\sigma^{i}\right)$ = $\sigma^{i-1}$ 12:    **Return** 13: **End if** 14: **If** there is no aggregator whose $S_{\sigma^{i-1}}$ is equal to $S_{\sigma^{i}}$ 15:    $\mathrm{index}=S_{\sigma^{i}}-1$ 16:    **While**
index>0
**do** 17:        **For** each $S_{\sigma^{i-1}}$
**do** 18:            **If**
$S_{\sigma^{i-1}}==\mathrm{index}$
**then** 19:                $Dest\left(\sigma^{i}\right)=\sigma^{i-1}$ 20:                **Return** 21:            **End if** 22:        **End for** 23:        index-- 24:    **End while** 25:    $\mathrm{index}=S_{\sigma^{i}}+1$ 26:    **While**
index≤Q
**do** 27:        **For** each $S_{\sigma^{i-1}}$
**do** 28:            **If**
$S_{\sigma^{i-1}}==\mathrm{index}$
**then** 29:                $Dest\left(\sigma^{i}\right)=\sigma^{i-1}$ 30:                **Return** 31:            **End if** 32:        **End for** 33:        index++ 34:    **End while** 35: **End if**


The explicative remarks about Algorithm 3 are shown below:

Lines 3–8: Aggregator searches an aggregator whose service tag is equal to the aggregator’s service tag.

Lines 9–13: The aggregator finds a neighbor aggregator with no service tag as its next hop and gives the tag to that aggregator.

Lines 15–24: If there is no aggregator owning the corresponding service tag or no tag, the aggregator searches an aggregator which has a better service from bad to good.

Lines 25–34: If there is no aggregator with no corresponding service or better or no tag, the aggregator starts to find an aggregator with a worse service from good to bad.

The complexity analysis is described below:

The number of the aggregators in the network is set as N. the time that aggregator broadcasts and receives message is O(1). Then the aggregator selects an aggregator with the corresponding tag. The overhead of this step is O(N). If there is no this kind of aggregator, the aggregator finds one aggregator with no tag. The cost is O(N). If there is no such an aggregator, the sensor would go through the tags twice at most. Therefore, the time complexity of an aggregator selects an aggregator is as Equation (8):

(8) O(1)+O(N)+O(N)+O(N)+O(N)=O(N)

*Phase 5:* Service tag rotation

The different service requirements in the network cause the different frequency of data aggregation and transmission. Aggregation and transmission are the primary energy consumption methods of nodes. Consequently, nodes frequently transmitting data packets consume energy fast. As a consequence, the early death of these nodes has. So service tag rotation needs to be adopted in DDAR. Each aggregator uses the tag opposite to the current tag as its service tag (for example, there are five service requirements, the current tag of node σ is 1, the tag after tag rotation is 5), the operations phase 2 to phase 4 would be performed again after the rotation.

## 5. Performance Analysis and Optimization

Considering the aggregation threshold, the aggregation timer, the data aggregation ratio, the probability and the service requirement as the dominant parameters that determine the performance of the data aggregation during the transmission process, simulation experiments implemented by C++ programing language in different scenarios are conducted in this section.

The density of nodes and the number of layers do not affect the performance of networks. Some experiments are designed, and the service guarantee rate of each network are compared. Table 4 proves that the structure of network does not affect the performance. Thus, the structure parameters of the network in simulations are constant, the radius of the network is 100 m, the radius of transmission of all nodes is 20 m and the number of nodes in the network is 1000.

In a scenario of the simulation, the number of the type of service requirements and the value of each requirement are predetermined, requirements are randomly assigned to the nodes. The averages are calculated based on the value of predetermined requirements. The average gap is the average difference between the value of requirements in a scenario. To present the scalability of DDAR, the environment parameters are shown in Table 5. A set of service requirements are described as {Q, average v(Q), average gap of each Q}.

In a simulation, 200,000 packets are generated in the whole network. 200 simulations are conducted for the same setting and get the average value of each indicator. To demonstrate the optimization effect, the performance of DDAR is compared to the CDAR network with the same environment. The Common Data Aggregation Routing (CDAR) scheme is that: in CDAR scheme, all the aggregators are homogenous in aggregation threshold and aggregation timer, which means that there is no differentiated aggregation routing, data packets with different service requirements are handled as the homogenous data packets.

We first study the performance of the optimization in various scenarios regarding service guarantee rate in Section 5.1. In Section 5.2, we study the optimization performance regarding network lifetime. The optimization on energy consumption is shown in Section 5.3. Finally, we compare the performance of the common DDAR scheme and the improved DDAR scheme in Section 5.4.

### 5.1. Optimization Performance on Service Guarantee Rate

In this subsection, we aim to investigate the optimization effect of the DDAR scheme in different circumstances. The optimization regarding service guarantee rate is shown in this subsection.

From Table 6, it can be seen that the optimization performance exists in all networks. The ratio of ρ is the average value of ρ in DDAR scheme divide the value of ρ in CDAR scheme with different value of $P_{\alpha }$, λ and η. DDAR scheme shows a better performance which the service guarantee rate increases by 25.01% on average. 

**Figure 9:**
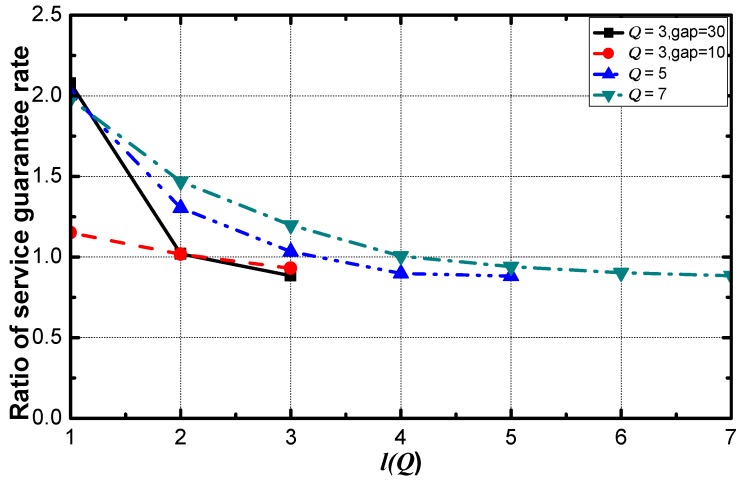
The ratio of service guarantee rate under different cases.

To find the parameter that dominates the performance of optimization, the networks whose service requirement is {3, 50, 10} is added in the simulation. As can be seen in Figure 9, the ratio decreases with the value of requirement increases and is saturated at 0.88 eventually. The performance of the network with the average gap of 10 is worse than that of the network with the average gap of 30. The added scenario shows that with the same number of requirements, the larger the average gap the service requirements, the better performance DDAR scheme has. In fact, the optimization performance is determined by the average gap of the requirements. If the requirement is smaller than the average requirement, there is a positive optimization effect. And if the requirement is larger than the average, there is a negative impact on optimization. Larger the absolute gap value, more obvious the impact of optimization. Additionally, the positive impact is obviously greater than the negative impact when the absolute value of gap is the same, this is the reason that DDAR performs better. Therefore, the parameter that determines the optimization performance is the average value of the difference between the average value of requirement and each value of requirement. DDAR has a good performance when the network has a large average differences of service requirements.

**Figure 10:**
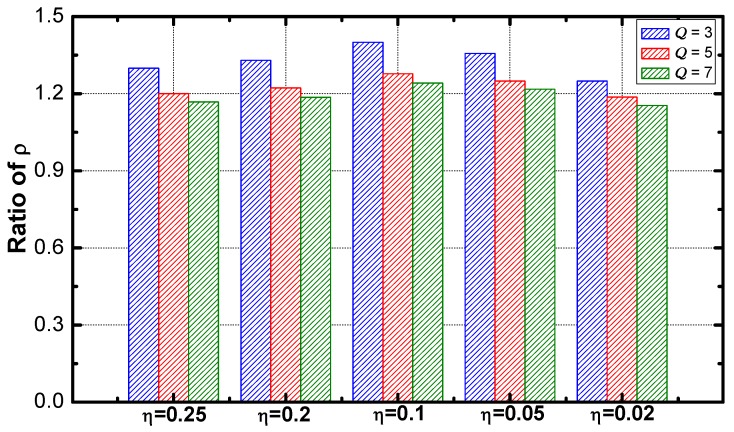
Average ρ in the network with different η.

Figure 10 demonstrates the optimization regarding ρ under the environments with a different value of η. With the increase of η, the optimization performance increases and decreases slightly. The networks with a η value of 0.1 have the best performance.

**Figure 11:**
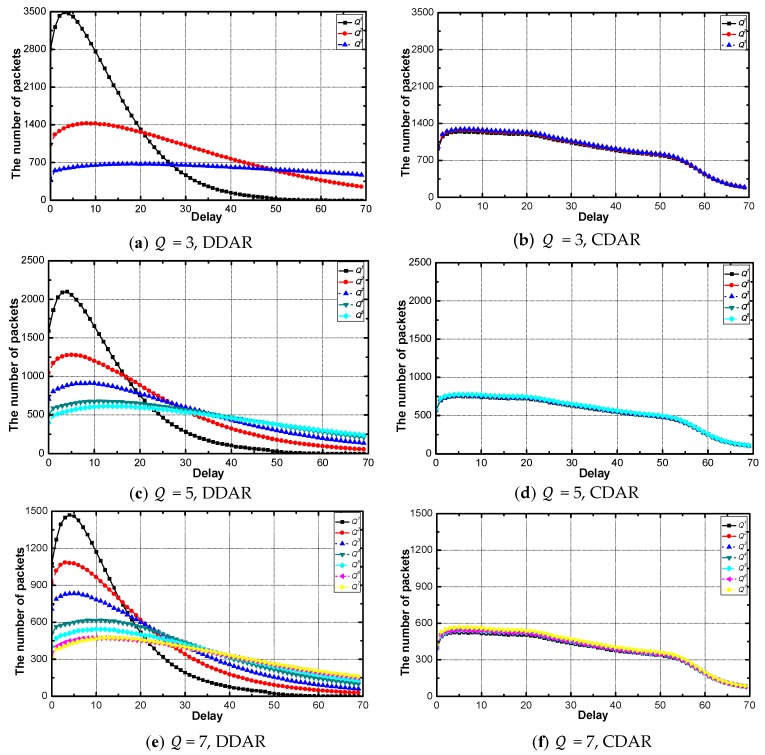
The distribution of delay when $P_{\alpha }$ = 0.5, λ = 0.6, η = 0.25.

Figure 11 shows the delay distribution of the data packets with each service requirement under the networks with various Q. The number of data packets increases rapidly at first and gradually decreases to 0. In the networks with DDAR, the networks with different Q have different delay distributions. The smaller the delay requirement, the more packets concentrated toward the small delay part and more sharply the number rises and falls. The packet delay distributions are the same for all Q in all networks with CDAR.

### 5.2. Optimization Performances on Lifetime

The death the time that the first node’s death occurs in the network is the lifetime of the network, the energy consumption rate of an aggregator which die first is inversely proportional to the lifetime. In data aggregation and transmission process, the energy consumption is directly proportional to the length of data packets queue. In the simulation, the energy consumption of aggregating and transmitting a data packet is set as 0.01 Joule and the energy to start aggregation and transmission is 0.2 Joule.

(a) Overview

The maximum energy consumption under various environments is listed in Table 7. The average energy consumption of the networks adopting DDAR is much smaller than that of the networks adopting CDAR. The performance of optimization on lifetime is immune to the change of Q.

**Figure 12:**
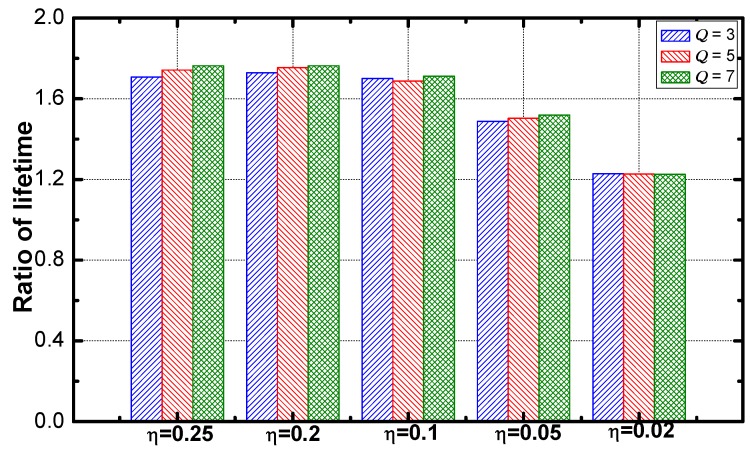
The ratio of lifetime vs. η.

As seen from Figure 12, the optimization performance disappears as η decreases. And the performance is not affected by the change of the difference of the service requirements.

(b) The effect of $P_{\alpha }$ and λ

From Table 8 we can find that the maximum energy consumption decreases with the increase of $P_{\alpha }$ in the networks with CDAR, and the performance is not affected by the change of $P_{\alpha }$. Therefore, the optimization effect regarding lifetime decreases slightly as $P_{\alpha }$ increases.

**Figure 13:**
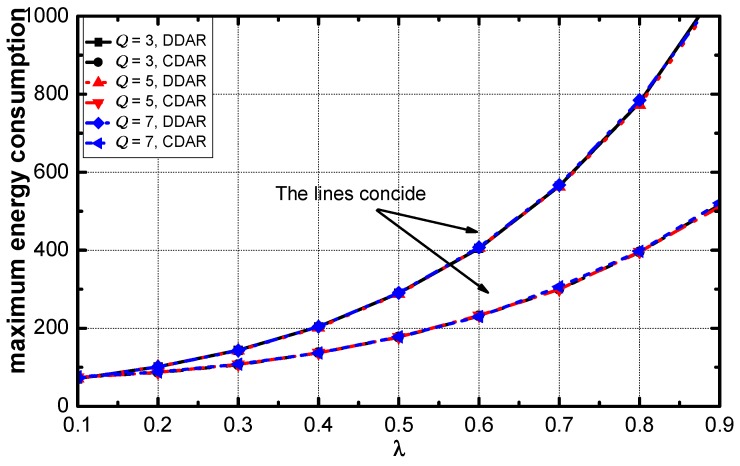
Average maximum energy consumption vs. λ.

Figure 13 illustrates that the maximum energy consumption in the networks with DDAR and CDAR and the difference of maximum energy consumption increases with the increase of λ. The maximum energy consumption in the networks of these two schemes increases exponentially.

**Figure 14:**
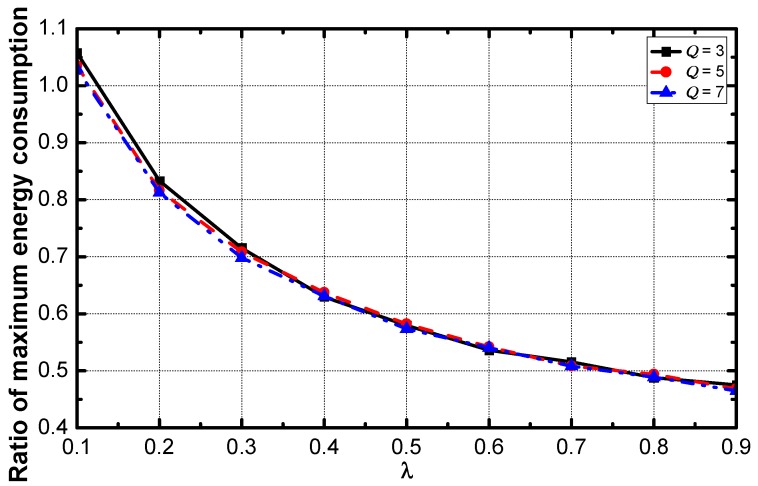
The average ratio of maximum energy consumption vs. λ.

Since the energy consumed in the network using CDAR increases faster than the energy consumed in DDAR networks, the ratio of the consumption decreases with the increase of λ (see from Figure 14). In the networks with a large value of λ, the lifetime can be prolonged to twice. When the value of λ is small than 0.1, the negative optimization effect appears.

### 5.3. Optimization Performance on Energy Efficiency

In this subsection, we mainly deal with the optimization performance of DDAR scheme regarding energy efficiency. Energy efficiency is defined as the ratio of the sum of energy consumption of all the aggregators when an aggregator dies in the network to the sum of the energy of all aggregators. 

(a) Overview

Table 9 shows the average energy efficiency and the ratio of energy efficiency under various environments. DDAR significantly improves the energy efficiency. The improvement does not change with the change of Q.

**Figure 15:**
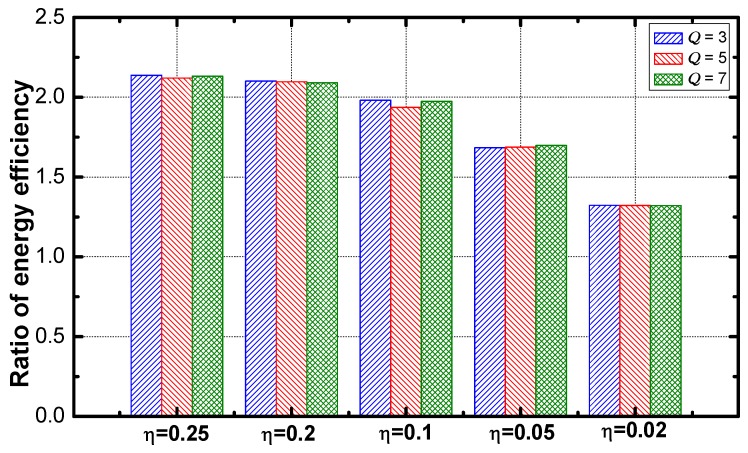
Ratio energy efficiency vs. η.

It can be seen in Figure 15 that the optimization effect increases when η increases.

(b) The effect of $P_{\alpha }$ and λ

In Table 10, the energy efficiency in CDAR and DDAR is not affected by the change of Q. The energy efficiency decreases as $P_{\alpha }$ increases in the networks with DDAR, but it decreases more slightly in CDAR networks. Therefore, the optimization performance decreases with the increase of $P_{\alpha }$.

**Figure 16:**
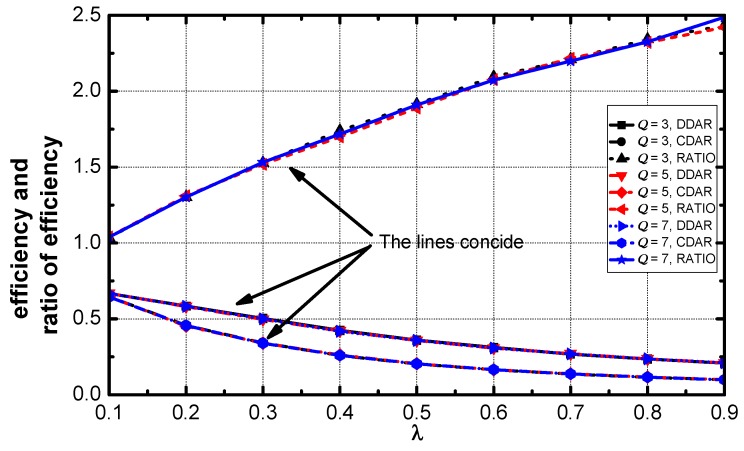
Average energy consumption and the ratio vs. λ.

As revealed in Figure 16, the efficiency decreases as the value of λ increases. The ratio approximately linearly increases with the increases of λ.

### 5.4. Performance of the Improved DDAR Scheme vs. the Common DDAR Scheme

In this subsection, we propose a strategy to improve the service guarantee rate, lifetime and energy efficiency of DDAR.

The value of $N_{t}$ and $T_{t}$ are set according to predetermined $N_{t}$($\bar{N_{t}}$) and predetermined $T_{t}$($\bar{T_{t}}$) calculated by the configuration formula in DDAR scheme. We design a formula to achieve the configuration of $N_{t}$ and $T_{t}$ for aggregators which need i hops to the sink, the formula has shown below (the formula to $T_{t}$ is the same):

(9) $$N_{t}=\{\begin{array}{l} \bar{N_{t}}\frac{L-i{\left(\log_{2}\frac{1}{\lambda }\right)}^{3}}{L},\;\lambda <0.5\\ \bar{N_{t}}\frac{L-i}{L}\log_{2}\frac{1}{\lambda },\;\lambda \geq 0.5 \end{array}$$

Service guarantee rate, lifetime and energy efficiency of the networks adopting the improved DDAR scheme are compared with those of the networks adopting the common DDAR. The results are listed in Table 11. With the 3% increase in maximum energy consumption, date efficiency increases 33.97% and service guarantee rate increases 10.11%.

**Figure 17:**
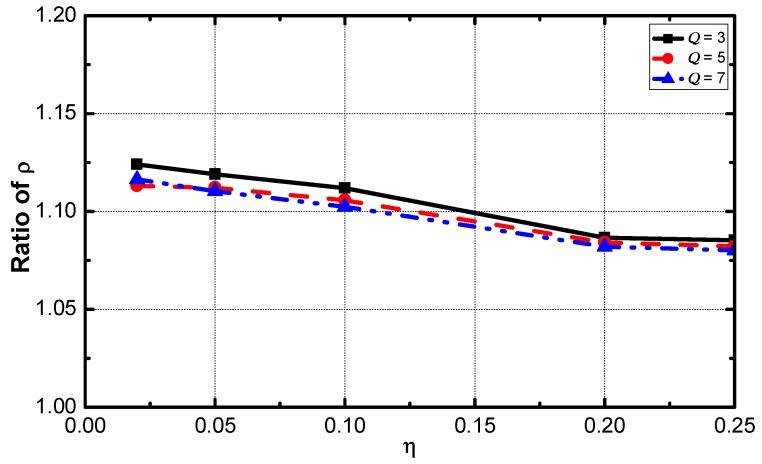
The ratio of service guarantee rate vs. η.

**Figure 18:**
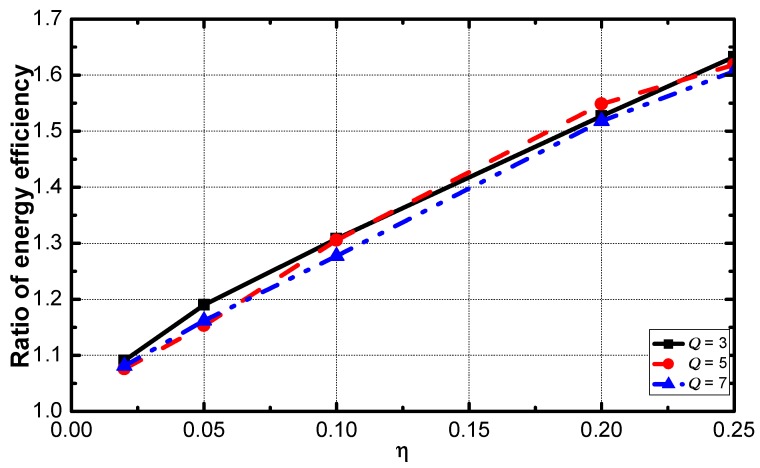
The ratio of energy efficiency vs. η.

**Figure 19:**
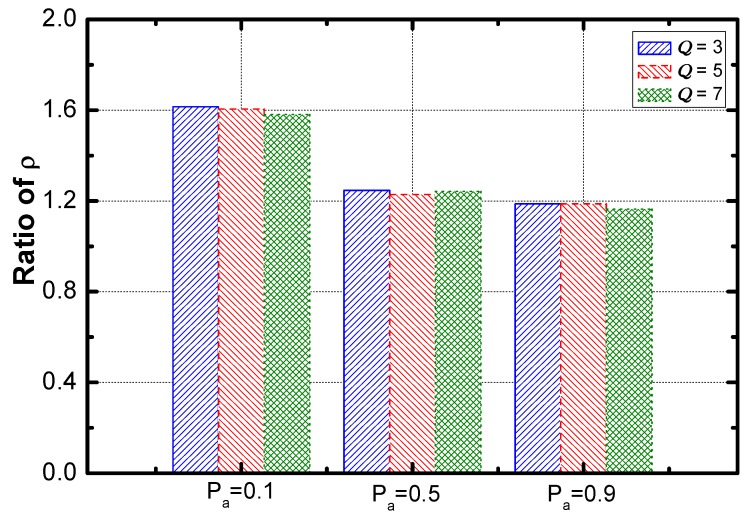
The ratio of service guarantee rate vs. $P_{\alpha }$.

Figure 17 shows that the optimization effect slightly decreases as the value of η increases. The service guarantee rate and energy efficiency are compared. As the results are shown in Figure 18 and Figure 19. The ratio of the rate decreases slightly as η increases and the ratio of energy efficiency increases linearly with the increase of η.

**Figure 20:**
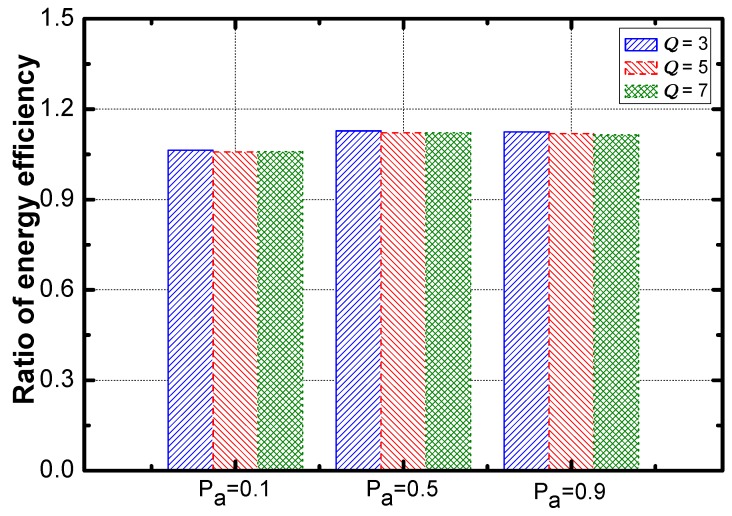
The ratio of energy efficiency vs. $P_{\alpha }$.

**Figure 21:**
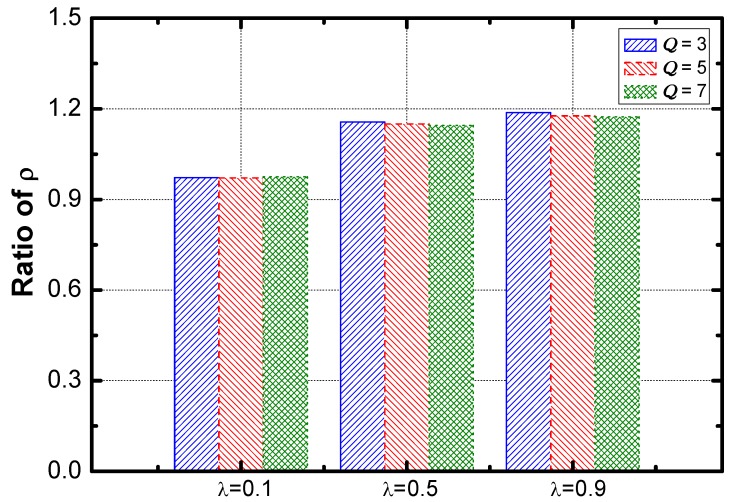
The ratio of service guarantee rate vs. λ.

Regarding the effect of $P_{\alpha }$, Figure 20 shows that the optimization of service guarantee rate decreases sharply and then is saturated. Figure 21 reveals that the optimization of energy efficiency increases slightly and then is saturated.

**Figure 22:**
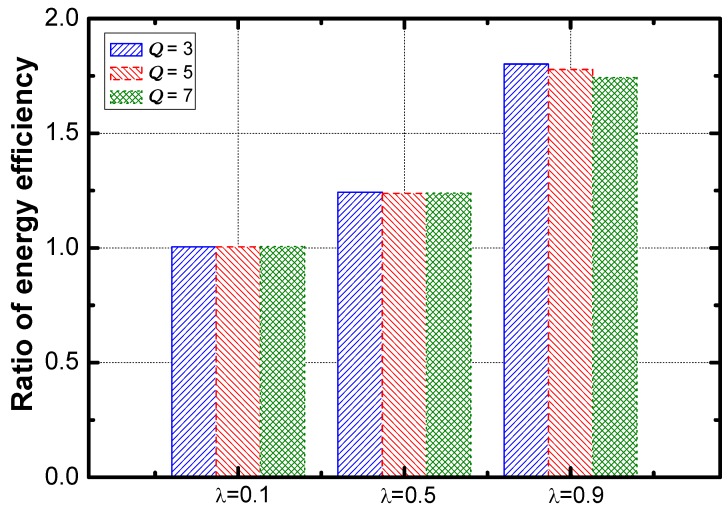
The ratio of energy efficiency vs. λ.

Here is the effect of the aggregation ratio. The optimization effect regarding energy efficiency is improved faster than that of service guarantee rate (as seen in Figure 21 and Figure 22).

## 6. Conclusions

In DiffServ networks, data have varying degrees of tolerance for delay. According to this feature, we propose a DDAR scheme to enhance the performance in DiffServ networks. DDAR scheme first sets service tag and configures $N_{t}$ and $T_{t}$ for all aggregators. Then the sensors select the aggregators that send their packets to according to the service requirements of the data packets. Meanwhile, aggregators select the aggregators in the upper layer with the same service tag as their next hop. During the data transmission, the aggregators periodically rotate the provided service to balance the energy consumption. In this paper, DDAR is compared with CDAR, and the performances of DDAR regarding service guarantee rate, network lifetime, and energy efficiency are studied. In the networks, the probability of generating a data pakcet, aggregation ratio, and the propotion of aggregators are the environment parameters that mainly affect the performances, extensive simlations are conducted by changing the values of these parameters. On the whole, DDAR scheme achieves a remarkable optimization effect on DiffServ networks. In the simulations, the service guarantee rate increases by 25.01% on average. The optimization is determined by the average difference between the service requirements. The optimization is improved with the increase of the difference. In terms of network environment parameters, there is little difference when η changes. The good optimization effect appears when $P_{\alpha }$ is large or λ is small or large. Network lifetime increases by 55.45% and energy efficiency increases by 83.99%. These two indicators do not change with changes in service requirements. They both increase with the increase of η or λ. With the increase of $P_{\alpha }$, the lifetime decreases slightly and the energy efficiency decreases. On the basis of DDAR, the improved DDAR is proposed for the problem of low energy utilization for the nodes far away from the sink. In the scheme, the value of $N_{t}$ and $T_{t}$ are narrowed to increase the aggregation frequency. The results indicate that the improved DDAR improved the service guarantee rate and energy efficiency by 10.11% and 33.97% respectively without degrading the lifetime.

DDAR starts with the specific delay requirements and determines the relevant parameters during the transmission according to the nature of the network, thereby ensuring the data validity of various services. It is believed that DDAR has an optimization effect in the DiffServ networks with same logical structure(e.g., cluster networks). We hope that more inspirations can be stimulated by our idea of the combination of the specific requirements and the nature of the networks and our research can contribute to the future works.

## Figures and Tables

**Table 1:** The references summary of research on data aggregation routing.

Problem Set	Work
Convergecast	Xu et al. [41], Huang et al. [58]
Adaptive Aggregation Scheme	Li et al. [42]
Optimize Data Aggregation	Villa et al. [61]
Cluster-based WSNs	Nazhad et al. [62]
Approximate Routing	Liu et al. [63]

**Table 2:** The references summary of research on delay optimization.

Problem Set	Work
Optimizing the transmission power	Xu et al. [16]
Routing Algorithms to Balance The Energy	Huang et al. [20], Tang et al. [36], Naranjo et al. [38], Xu et al. [39], Hazard et al. [57]
Optimizations Including Security	Tang et al. [36]
Data packets split	Liu et al. [63]
Optimizing The Delay in Duty Cycle	Liu et al. [64]
Sending Data to Multiple receivers	Xu et al. [39],
Optimization for Retransmission	Liu et al. [65]

**Table 3:** System parameters.

Parameter	Description
N	The number of the nodes in the network.
L	The number of the layers in the network.
η	The proportion of aggregators in nodes.
σ	An aggregator.
ε	A sensor.
$N_{t}^{i}$	The value of packet aggregation threshold for the service requirement i.
$T_{t}^{i}$	The value of the packet aggregation timer for service requirement i.
$P_{\alpha }$	The probability that a sensor generates a data packet during a packet generation period.
λ	Data aggregation ratio.
Q	The number of the type of service requirements in a network.
$E_{INI}^{i}$	The initial energy in an aggregator i.
$\xi_{\sigma }$	The energy consumption of aggregator σ in a unit time
$l\left(Q_{\varepsilon }\right)$	The level of service requirement of sensor ε.
$v(Q^{i})$	The value of service requirement i.
ρ	Service guarantee rate.
$S_{\sigma }$	The service tag of aggregator σ.
Dest(ε)	The aggregator that sensor ε transmits the data packets to.
Dest(σ)	The next hop that the aggregated queues of aggregator σ transmits the data packets to.

**Table 4:** Service guarantee rate (%).

	*L* = 4	*L* = 5	*L* = 6	*L* = 7
N/L=10	97.61	97.66	97.73	97.71
N/L=30	97.53	97.66	97.81	97.74
N/L=50	97.65	97.73	98.02	98.42

**Table 5:** Simulation parameters.

Parameter	Value
R	100 (m)
r	20 (m)
N	1000
$P_{\alpha }$	{0.1, 0.5, 0.9}
λ	{0.1, 0.2, 0.3, 0.4, 0.5, 0.6, 0.7, 0.8, 0.9}
η	{0.02, 0.05, 0.1, 0.2, 0.25}
Q	{3, 5, 7}
Q	{3, 50, 30},{5, 50, 15},{7, 50, 10}

**Table 6:** Optimization of service guarantee rate.

	Q=3	Q=5	Q=7
Ratio of ρ (%)	132.7	122.65	119.68

**Table 7:** Average maximum energy consumption and the ratio in each environment.

	DDAR (J)	CDAR (J)	Ratio (%)
Q=3	226.78	403.57	64.78
Q=5	224.82	400.70	64.35
Q=7	224.79	403.86	63.86

**Table 8:** Average maximum energy consumption and the ratio vs. $P_{\alpha }$.

	$P_{\alpha }=0.1$	$P_{\alpha }=0.5$	$P_{\alpha }=0.9$
DDAR (J)			
Q=3	229.41	224.38	229.54
Q=5	229.55	216.62	228.28
Q=7	229.09	218.42	226.87
CDAR (J)			
Q=3	423.44	396.69	390.58
Q=5	420.37	396.96	384.78
Q=7	422.43	398.75	390.40
Ratio (%)			
Q=3	63.53	63.97	67.85
Q=5	63.30	62.18	67.63
Q=7	62.72	61.90	66.98

**Table 9:** Average energy efficiency and the ratio in each environment.

	DDAR (%)	CDAR (%)	Ratio (%)
Q=3	39.60	26.94	184.49
Q=5	39.47	26.97	183.22
Q=7	39.45	26.92	184.26

**Table 10:** Average energy efficiency and the ratio vs. $P_{\alpha }$.

	$P_{\alpha }=0.1$	$P_{\alpha }=0.5$	$P_{\alpha }=0.9$
DDAR (%)			
Q=3	47.65	37.19	33.96
Q=5	47.00	37.35	34.05
Q=7	46.81	37.32	34.22
CDAR (%)			
Q=3	29.55	25.73	25.96
Q=5	29.58	25.64	25.62
Q=7	29.56	25.64	25.78
Ratio (%)			
Q=3	199.98	184.03	169.46
Q=5	195.24	185.24	169.91
Q=7	194.94	185.67	172.17

**Table 11:** Optimization of energy efficiency.

Scenario	Energy Efficiency (%)	Service Guarantee Rate (%)	Energy Consumption (%)
Q=3	134.97	110.54	102.88
Q=5	134.03	109.95	103.23
Q=7	132.90	109.83	103.44
